# NMDA receptor involvement in dopaminergic modulation of neuroplasticity induced by paired associative stimulation

**DOI:** 10.1093/ijnp/pyaf038

**Published:** 2025-05-30

**Authors:** Marie C Beaupain, Elham Ghanavati, Amba M Frese, Lorena Melo, Min-Fang Kuo, Michael A Nitsche

**Affiliations:** Department of Psychology and Neurosciences, Leibniz Research Centre for Working Environment and Human Factors (IfADo), Dortmund, Germany; Department of Psychology, Ruhr-University Bochum, Bochum, Germany; Department of Psychology and Neurosciences, Leibniz Research Centre for Working Environment and Human Factors (IfADo), Dortmund, Germany; Department of Psychology, Ruhr-University Bochum, Bochum, Germany; Department of Psychology and Neurosciences, Leibniz Research Centre for Working Environment and Human Factors (IfADo), Dortmund, Germany; International Graduate School of Neuroscience (IGSN), Ruhr-University Bochum, Bochum, Germany; Rehabilitation Research Center, Faculty of Rehabilitation Sciences, University of Hasselt, Diepenbeek, Belgium; Department of Psychology and Neurosciences, Leibniz Research Centre for Working Environment and Human Factors (IfADo), Dortmund, Germany; Department of Psychology and Neurosciences, Leibniz Research Centre for Working Environment and Human Factors (IfADo), Dortmund, Germany; Department of Psychology and Neurosciences, Leibniz Research Centre for Working Environment and Human Factors (IfADo), Dortmund, Germany; University Clinic of Psychiatry and Psychotherapy Protestant Hospital of Bethel Foundation, University Hospital OWL, Bielefeld University, Bielefeld, Germany; German Center for Mental Health (DZPG), Bochum/Marburg, Germany

**Keywords:** neuroplasticity, dopamine, NMDA receptor, paired associative stimulation

## Abstract

**Background:**

Dopamine (DA) modulates long-term potentiation (LTP)-like neuroplasticity. While particularly D1 and D2 receptors are thought to influence neuroplasticity through glutamatergic N-methyl-D-aspartate (NMDA) receptor and gamma-aminobutyric acid (GABA) modulation, the exact mechanisms are not completely clarified.

**Objective:**

We aimed to explore the relevance of NMDA receptor activity for DAergic modulation of focal LTP-like plasticity induced by excitatory paired associative stimulation (ePAS).

**Methods:**

In a double-blinded, randomized, and placebo-controlled design, 17 healthy participants received DAergic agents (100 mg L-Dopa for general DAergic enhancement, 10 mg bromocriptine for selective D2 receptor activation, or placebo) with different doses of the partial NMDA receptor agonist D-cycloserine (CYC; 50, 100, 200 mg, or placebo) and underwent ePAS. Cortical excitability was monitored via motor-evoked potentials induced by TMS over the left motor cortex for up to 2 hours post-stimulation.

**Results:**

We did not find significant interactions between DAergic agents, CYC, and time across the entire sample, but significant group differences depending on sensitivity to ePAS. In high-sensitivity, but not low-sensitivity participants, ePAS induced LTP-like effects. CYC produced nonlinear, dose-dependent effects on plasticity in both groups. In the high-sensitivity group, LTP-like effects persisted under both DAergic agents, but were significantly reduced under bromocriptine. CYC had a nonlinear effect when combined with bromocriptine. In the low-sensitivity group, ePAS under DAergic agents did not induce LTP-like effects, and only additional intervention with medium-dose CYC restored facilitatory effects under L-Dopa.

**Conclusions:**

These findings suggest that optimal NMDA receptor activation is necessary for ePAS-induced neuroplasticity and that D2 receptor activity may reduce LTP-like effects by downregulating NMDA receptor function.

**Significance Statement**

Long-term potentiation (LTP)-like neuroplasticity is central to cognitive processes, such as learning and memory formation, and its impairment can be observed in mental disorders, such as schizophrenia or depression. Dopamine, particularly through D2 receptor activity, plays a key role in modulating LTP-like neuroplasticity. This study shows that general dopaminergic and D2 receptor activity affect focal LTP-like plasticity induced by excitatory paired associative stimulation in the motor cortex through modulation of glutamatergic N-methyl-D-aspartate (NMDA) receptor activity. Our findings provide insight into the neurophysiological mechanisms underlying dopaminergic and NMDA receptor-related effects on neuroplasticity in healthy individuals and may inform clinical applications of pharmacological and brain stimulation interventions in dopamine-associated conditions, such as attention-deficit hyperactivity disorder or schizophrenia.

## INTRODUCTION

Dopamine (DA) is central to core brain functions, such as motor control and memory; DAergic imbalance is associated with conditions, such as Parkinson’s disease and schizophrenia.^1,2^ The neuromodulator DA affects the extent and direction of long-term potentiation (LTP) and long-term depression (LTD),^3,4^ which presumably form the cellular basis of learning processes.^5^ Previous research suggests a “focusing effect” of DA by enhancing the signal-to-noise ratio of neural processing by increasing relevant and decreasing irrelevant activity.^4^

Brain stimulation techniques used to investigate the focusing effect of DA include paired associative stimulation (PAS) and transcranial direct current stimulation (tDCS). PAS pairs electrical stimulation of a peripheral somatosensory afferent with cortical brain stimulation via transcranial magnetic stimulation (TMS).^6^ If afferent stimulation precedes the TMS pulse by 25 ms, both impulses reach the cortical target near-synchronously, inducing excitatory neuroplastic effects (ePAS). Since PAS affects specific sensorimotor connections and is spatially limited to these, the induced changes are more focal than those generated by tDCS, which modulates the resting membrane potential of larger neuronal populations on a subthreshold level independent of connectivity.^7,8^ As PAS functions on a suprathreshold level and triggers action potentials, it induces stronger neuronal activation.^9,10^ For both stimulation techniques, plasticity effects depend on neuronal calcium (Ca2^+^) influx and the activity of glutamatergic N-methyl-D-aspartate (NMDA) receptors.^7,11,12^

Consistent with a focusing effect of DA, medium doses of the DA precursor L-Dopa prolonged,^8^ or prolonged and enhanced^4^ LTP-like neuroplasticity induced by focal ePAS, but abolished LTP-like effects induced by diffuse anodal tDCS or converted these into LTD-like effects in previous studies.^4,13^ Similarly, medium doses of the D2 receptor agonist bromocriptine abolished LTP-like plasticity induced by tDCS,^13,14^ while these effects were largely preserved under ePAS, although to a reduced extent.^,^^14^ Similar effect patterns under general DA enhancement and selective D2 receptor activation lead to the assumption that especially D2 receptors may contribute to the focusing effect of DA in the healthy human brain. The co-application of the partial NMDA receptor agonist D-cycloserine (CYC) in a medium dose restored tDCS-induced LTP-like plasticity, which had been converted or abolished under L-Dopa or bromocriptine,^13^ in accordance with the suggestion that D2 receptor activation reduces irrelevant neural activity via reduced NMDA receptor activity and neuronal Ca2^+^ influx.^15^

Differences between stimulation protocols constitute the focusing effect of DA, and might involve prolongation or enhancement of the magnitude, but also lead to diminution, abolishment, or conversion of plasticity effects. Therefore, this study examines potential D2 receptor contributions to NMDA receptor-dependent DAergic modulation of LTP-like plasticity induced by suprathreshold, focal ePAS. We combine medium doses of globally DA-enhancing L-Dopa or the selective D2 receptor agonist bromocriptine with a low, medium, or high dose of the partial NMDA receptor agonist CYC and respective placebo conditions, and apply ePAS to induce focal LTP-like plasticity in the motor cortex. Based on previous observations, we expect stabilized LTP-like plasticity under general DA enhancement, and reduced, but not abolished LTP-like plasticity under D2 receptor enhancement, due to the more focal and stronger stimulation accomplished by ePAS. We hypothesize that adding CYC enhances DA-reduced NMDA receptor activity and dose-dependently restores LTP-like effects.

## METHODS

### Participants

We recruited 19 right-handed, nonsmoking healthy participants. Two dropped out after experiencing side effects from applied substances. Therefore, data of 17 participants were analyzed (9 female, mean age: 27.24, range: 19–35 years, mean laterality quotient: 89.65, SD: 10.28, according to Oldfield^16^). A medical screening confirmed the absence of a history of neurological or psychiatric disorders, intake of CNS-affecting medication, metal implants, and potential pregnancy. The minimum sample size was determined based on previous neuropharmacological studies.^17,18^ For critical alpha and beta errors of 0.05 and a medium effect size of *f* = 0.35, the power calculation for a repeated-measures ANOVA resulted in a sample size of 10 subjects. The final sample size of *N* = 17 was therefore adequate. The study was performed according to the guidelines of the Declaration of Helsinki and approved by the Institutional Review Board of the Leibniz Research Centre for Working Environment and Human Factors (Dortmund, Germany). All participants gave their written informed consent and received monetary compensation for participation.

### Pharmacological Interventions

Participants underwent 12 pharmacological conditions in a double-blinded, randomized, and placebo-controlled experimental design. We applied 3 DAergic conditions: 100 mg of L-Dopa in combination with 25 mg of the DA decarboxylase inhibitor benserazide (L-Dopa/Benserazid—Neuraxpharm; peak concentration after 1.5 hours); 10 mg of bromocriptine (Parlodel, Mylan Medical SAS; peak concentration after 2 hours), or an equivalent placebo (P pills, Zentiva Pharma GmbH), and combined these with 4 doses of the NMDA-receptor agonist D-cycloserine (50, 100, 200 mg—Meiji, or equivalent placebo; peak concentration after 2 hours). While L-Dopa enhances general DA levels, bromocriptine selectively increases D2 receptor activity. Both DAergic agents in the chosen medium doses have affected ePAS-induced facilitatory plasticity in previous studies.^4,8,14^ To reduce systemic side effects of DAergic substances, participants took 20 mg of domperidone (Hexal) 3 times per day for 2 days prior to each experimental session, and 1 dose 2 hours before substance intake. Domperidone itself does not affect motor cortical excitability.^19^

### Transcranial Magnetic Stimulation

TMS was applied through a figure-of-eight coil (diameter of one winding: 70 mm; peak magnetic field: 2 T) connected to a PowerMag stimulator (Mag&More). Each experimental session started with placing the coil tangentially to the skull at a 45° angle to the sagittal plane over the left primary motor cortex and eliciting motor-evoked potentials (MEPs) in the right abductor digiti minimi muscle (ADM), measured by surface electrodes positioned in a belly-tendon montage over the ADM. The optimal stimulation position was identified as the spot where TMS pulses, delivered at medium intensity, consistently produced the largest MEPs. To ensure identical positioning of the TMS coil throughout the experiment, the hotspot was marked with a soft tip pen on the scalp. Stimulation intensity was set to the percentage of maximum stimulator output (%MSO) required to elicit MEPs with an average peak-to-peak amplitude of 1 mV in 25 TMS pulses (SI1mV). Raw signals were amplified and filtered (1000; 3 Hz–3 kHz) with a D440-2 (Digitimer) with a time constant of 10 ms and a low-pass filter of 2.5 kHz. MEPs were then digitized with a micro 1401 AD converter (Cambridge Electronic Design) at an analog-to-digital rate of 5 kHz, controlled and recorded by Signal software (version 7.00, Cambridge Electronic Design).

### Induction of Neuroplasticity by PAS

Peripheral nerve stimulation was delivered to the right ulnar nerve at wrist level through a bipolar electrode (cathode proximal) with a Digitimer D185 stimulator (Digitimer). The stimulation intensity was set to 300% of the perceptual threshold, the latter defined as the lowest intensity perceivable by the participant. During PAS, a peripheral pulse was followed by a single TMS pulse over the motor cortex hotspot with SI1mV. We applied a stimulation protocol of 90 paired peripheral and TMS pulses at 0.05 Hz over 30 minutes with an interval of 25 ms, which is suited to induce LTP-like neuroplasticity in the motor cortex.^6^

### Experimental Procedure

**Figure 1.**
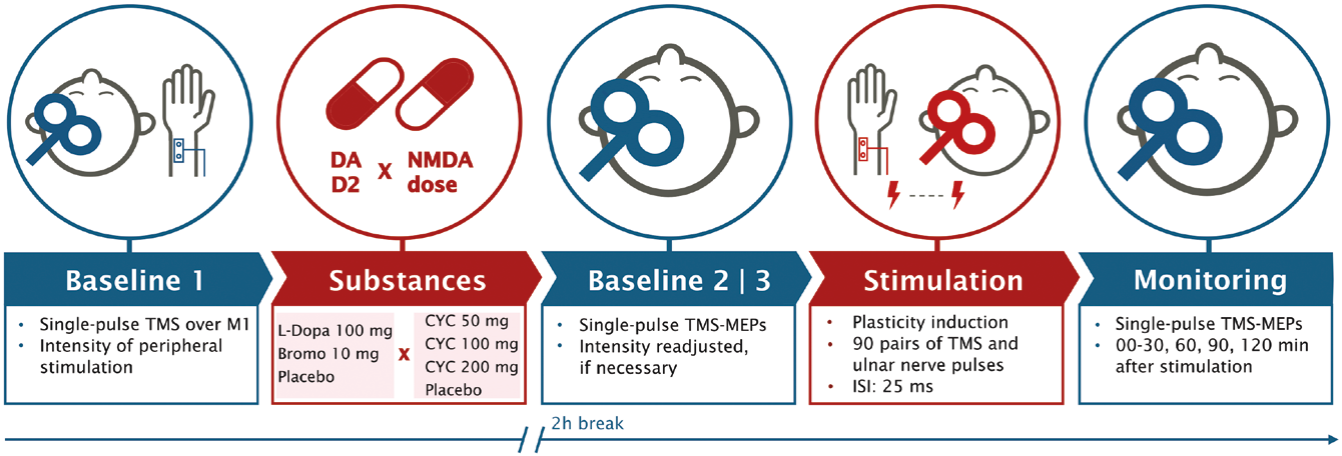
Timeline of the experimental sessions. After determining stimulation parameters for TMS and peripheral nerve stimulation, a baseline of 25 MEPs was recorded, followed by substance administration. After 2 hours, baselines 2/3 were recorded shortly before starting the ePAS protocol. Aftereffects were monitored every 5 minutes for 30 minutes, and every 30 minutes until 120 minutes post-PAS. Bromo, bromocriptine, CYC, D-cycloserine; ISI, inter-stimulus interval; MEP, motor-evoked potential; TMS, transcranial magnetic stimulation.

Participants returned for 12 experimental sessions with an intersession interval of at least 7 days to avoid cumulative substance or stimulation effects. They were seated in a reclining chair throughout the experiments, with their head placed on a vacuum cushion. After assessing stimulation location and intensity, a first TMS baseline (BL1) consisting of 25 consecutive single pulses with SI1mV intensity was recorded (see Figure 1). Electrode position and stimulation intensity for peripheral stimulation were determined before administering the experimental substance. PAS started 2 hours after oral intake of the test substances to ensure plasticity induction during peak plasma concentration.^14,20,21^ Following this timeline has resulted in prominent effects of these substances on plasticity before.^8,13,14,22^ A second TMS baseline (BL2) was measured shortly before PAS to control for substance effects on cortical excitability. If the mean of BL2 deviated more than 20% from BL1, stimulation intensity was adjusted to result in a mean MEP amplitude of 1 mV, and another 25 MEPs were recorded (BL3). To avoid attention fluctuations,^23^ participants were asked to focus on their relaxed right hand during PAS and count the MEPs. After-measurements consisted of 25 single pulse TMS-elicited MEPs at 0, 5, 10, 15, 20, 25, 30, 60, 90, and 120 minutes post-PAS. At the end of each session, participants were asked to freely report any side effects they experienced from the substances and rate their intensity on a scale from 1 (“very weak”) to 4 (“very strong”). They also completed a questionnaire on side effects from stimulation,^24^ rating their intensity on a scale from 0 (“absent”) to 4 (“very strong”).

### Statistical Analyses

Side-effect ratings were analyzed in 2 one-way repeated-measures ANOVAs with experimental condition as within-subject factor. MEPs with confounding muscle activity were removed after visual inspection, along with those deviating more than ±3-SD from the individual mean of each MEP block (1.33% of data removed). Mean peak-to-peak MEP amplitudes from post-stimulation measurements for each participant were normalized to individual BL2 or BL3 values, the latter if readjusted after substance application. Five 1-way ANOVAs with the within-subject factor experimental condition (12 levels) explored between-session baseline differences of peripheral stimulation, and TMS intensity, as well as absolute MEP amplitude values for BL1 and BL2 (or BL3) respectively. Two 2-way repeated-measures ANOVAs with the factors experimental condition (12 levels) and time (2 levels: pre- and post-substance) on MEP amplitudes of BL1 and BL2, as well as stimulation intensities of BL1 and BL3 evaluated if substances alone affected cortical excitability. To test substance effects on ePAS-induced neuroplasticity, we calculated a 3-way repeated-measures ANOVA with the within-subject factors DAergic agents (3 levels: L-Dopa, bromocriptine, placebo), CYC (4 levels: 50, 100, 200 mg, placebo), and time (11 levels: all measurements post-stimulation) on MEP amplitudes. In an additional analysis, we conducted a mixed-design ANOVA with gender as a between-subject factor based on the three-way repeated-measures ANOVA.

### Secondary Sensitivity Analyses

For the whole-group analyses, we noticed high interindividual variability in response to ePAS (grand average 0–30 minutes post-stimulation for the pure placebo substance session: *M *= 1.51, SD = 0.65, range: 0.62–3.14; see Table S1). To explore whether this affected the results, we calculated the average MEP amplitude from 0 to 30 minutes post-PAS for each participant in the pure placebo condition without real medication, since ePAS effects decreased or vanished after 30 min in previous studies.^4,8^ Based on this, we then determined the median MEP amplitude across our entire sample of 17 participants (median = 1.28), and classified our sample into 2 subgroups: “low ePAS sensitivity” (individual normalized mean MEP amplitude under pure placebo ≤ median, *n* = 9) and “high ePAS sensitivity” (individual normalized mean MEP amplitude under pure placebo > median, *n* = 8), and repeated the analyses described above to rule out group differences in stimulation intensities and baseline MEPs, and to explore substance effects on PAS-induced MEP amplitude changes. Differences in stimulation intensities and MEP amplitudes between groups were analyzed using Student’s independent *t*-tests (2-tailed) on mean data across experimental conditions. We performed an exploratory 3 × 4 × 2 × 2 mixed ANOVA with DAergic agents (L-Dopa, bromocriptine, placebo), CYC (50, 100, 200 mg, placebo), and time (baseline, grand average of 0–30 minutes post-stimulation) as within-subject factors, and ePAS sensitivity as between-subject factor. For significant ANOVA results, Student’s *t*-tests (2-tailed, not corrected for multiple comparisons) explored differences between sensitivity groups under pure placebo (2-sample *t*-test), and differences to baseline (1-sample *t*-tests, test value = 1), and between substance conditions and the placebo conditions (paired *t*-tests) within groups.

In the case of non-sphericity of ANOVA data (Mauchly’s test), we report *P*-values with Greenhouse–Geisser correction. The significance level was set at *P <* .05. Analyses were performed in SPSS (version 29.0), and figures were created in GraphPad Prism (version 10).

## RESULTS

### Adverse Effects

All participants tolerated stimulation well. Two participants experienced severe nausea after inconsistent domperidone intake and dropped out. In the remaining 17 participants, side effects were scarce and rated low to moderate in intensity (see Tables S2 and S3). Except for nausea, side effects were nondiscernible between experimental conditions (see Tables S4 and S5).

### No Differences in Baseline Measurements Between Experimental Conditions

The 1-way ANOVAs showed no significant baseline differences between the experimental conditions (see Table 1) for peripheral stimulation intensity (*F*_11,192_ = 0.221, *P* = .996), TMS intensity (BL1: *F*_11,192_ = 0.011, *P *= 1.000; BL2/3: *F*_11,192_ = 0.018, *P *= 1.000), or absolute MEP mean values (BL1: *F*_11,192_ = 0.184, *P* = .998; BL2/3: *F*_11,192_ = 0.555, *P* = .863). The substances alone did not affect cortical excitability (see Table S6).

**Table 1.** Baseline measurements: stimulation intensities and MEP amplitudes.

	TMS					Peripheral nerve stimulation
	SI 1 mV (%MSO)		MEP amplitude (mV)			SI (V)
	Baseline 1	Baseline 3	Baseline 1	Baseline 2	Baseline 3	
Placebo + Placebo	55.18 (10.68)	56.18 (12.14)	1.00 (0.07)	0.89 (0.23)	0.98 (0.09)	63.88 (14.10)
Placebo + 50 mg CYC	55.12 (11.01)	55.29 (11.21)	1.00 (0.06)	0.99 (0.15)	1.01 (0.13)	64.94 (14.19)
Placebo + 100 mg CYC	55.27 (10.88)	55.50 (10.77)	1.00 (0.07)	0.93 (0.20)	1.00 (0.10)	61.53 (12.16)
Placebo + 200 mg CYC	54.94 (12.03)	55.03 (12.04)	1.01 (0.06)	0.99 (0.26)	0.97 (0.09)	64.94 (12.86)
L-Dopa + Placebo	55.32 (11.08)	55.49 (11.24)	0.99 (0.07)	1.01 (0.16)	1.01 (0.11)	65.82 (13.10)
L-Dopa + 50 mg CYC	54.91 (11.64)	55.27 (11.36)	1.01 (0.05)	1.00 (0.15)	1.01 (0.08)	66.18 (13.73)
L-Dopa + 100 mg CYC	54.79 (10.90)	55.00 (11.17)	1.00 (0.07)	1.04 (0.32)	1.00 (0.09)	64.76 (15.04)
L-Dopa + 200 mg CYC	55.82 (11.04)	55.91 (10.77)	1.01 (0.08)	1.00 (0.21)	1.01 (0.09)	66.35 (11.85)
Bromo + Placebo	54.91 (10.72)	55.21 (10.79)	1.01 (0.04)	0.97 (0.10)	1.00 (0.07)	66.53 (12.52)
Bromo + 50 mg CYC	55.35 (12.10)	55.29 (12.15)	0.99 (0.08)	1.00 (0.13)	0.99 (0.11)	65.47 (12.29)
Bromo + 100 mg CYC	55.12 (11.04)	55.06 (11.16)	1.01 (0.05)	1.06 (0.23)	1.03 (0.09)	62.82 (11.10)
Bromo + 200 mg CYC	55.44 (11.79)	55.03 (11.81)	1.00 (0.07)	1.04 (0.19)	0.99 (0.10)	65.29 (13.23)

Abbreviations: Bromo, bromocriptine; CYC, D-cycloserine; MSO, maximum stimulator output; SI, stimulation intensity.

Data are presented as mean (*SD*).

### Results of the Whole-Group ANOVA for Pharmacological Intervention Effects on ePAS-Induced Plasticity

The 3-way repeated-measures ANOVA (DA × CYC × time, see Table 2) of normalized mean MEP amplitudes revealed significant main effects for DAergic agents (*F*_2,32_ = 7.544, *P* = .002) and time (*F*_2.7,43.2_ = 10.702, *P* < .001), but not for CYC. No significant interaction effects were present for DA × CYC, DA × time, CYC × time, or DA × CYC × time. See Figure S1 for visualization of mean MEPs across time per substance condition. No statistically significant gender effects were observed in this sample (see Table S7).

**Table 2.** Results of the 3-way repeated-measures ANOVA (DA × CYC × time).

	*df*	*F*	*P*	*η*_*p*_^*2*^
DA	2, 32	7.544	**.002**	0.320
CYC	2.1, 33.8	2.067	.140	0.114
time	2.7, 43.2	10.792	**<.001**	0.401
DA × CYC	3.6, 57.6	1.017	.401	0.060
DA × time	7.3, 116.5	1.791	.092	0.101
CYC × time	8.3, 133.1	1.348	.223	0.078
DA × CYC × time	9.3, 149.5	1.110	.359	0.065

Abbreviations: CYC, D-cycloserine (50, 100, and 200 mg, placebo); DA, dopaminergic agents (L-Dopa, bromocriptine, placebo); *df*, degrees of freedom; time: all 11 after-measurements; ηp^2^, partial eta-squared.

Bold *P* values indicate statistical significance.

### Results of the Secondary ANOVA Considering Sensitivity to ePAS

Stimulation intensities and baseline MEPs did not differ between experimental conditions in either low-sensitivity or high-sensitivity participants, and substances themselves did not affect cortical excitability in either group. The sensitivity groups did not differ in stimulation intensities or baseline MEP amplitudes (see Tables S8–S11).

The mixed 3 × 4 × 2 × 2 ANOVA revealed a significant main effect of ePAS sensitivity, indicating that MEP amplitudes differed significantly between sensitivity groups (*F*_1,15_ = 7.784, *P* = .014; see Table 3). DAergic agents had a significant main effect on MEPs (*F*_2,30_ = 3.914, *P* = .031), regardless of ePAS sensitivity. Significant main effects were present for CYC (*F*_3,45_ = 2.837, *P* = .049) and time (*F*_1,15_ = 7.679, *P* = .014), and depended on ePAS sensitivity (CYC × sens: *F*_3,45_ = 3.174, *P* = .033; time × sens: *F*_1,15_ = 7.784, *P* = .014). The nonsignificant interaction of DA × CYC varied across sensitivity groups (DA × CYC × sens: *F*_6,90_ = 2.850, *P* = .014), while the interaction of DA × time (*F*_2,30_ = 3.914, *P* = .031) did not. The combined effect of CYC × time (*F*_3,45_ = 2.837, *P* = .049) was different for sensitivity groups (CYC × time × sens: *F*_3,45_ = 3.174, *P* = .033). While there was no significant interaction of DA × CYC × time on a whole-group level, a significant interaction between these factors depending on sensitivity was observed (DA × CYC × time × sens: *F*_6,90_ = 2.850, *P* = .014).

**Table 3.** Results of the four-way mixed-design ANOVA (DA × CYC × time × ePAS sensitivity).

	*df*	*F*	*P*	*η*_*p*_^*2*^
Sens	1, 15	7.784	**.014**	0.342
DA	2, 30	3.914	**.031**	0.207
CYC	3, 45	2.837	**.049**	0.159
Time	1, 15	7.679	**.014**	0.339
DA × sens	2, 30	0.198	.822	0.013
CYC × sens	3, 45	3.174	.**033**	0.175
Time × sens	1, 15	7.784	**.014**	0.342
DA × CYC	6, 90	1.357	.285	0.077
DA × time	2, 30	3.914	**.031**	0.207
CYC × time	3, 45	2.837	**.049**	0.159
DA × CYC × sens	6, 90	2.850	**.014**	0.160
DA × time × sens	2, 30	0.198	.822	0.013
CYC × time × sens	3, 45	3.174	**.033**	0.175
DA × CYC × time	6, 90	1.257	.285	0.077
DA × CYC × time × sens	6, 90	2.850	**.014**	0.160

Abbreviations: CYC, D-cycloserine (50, 100, and 200 mg, placebo); DA, dopaminergic agents (L-Dopa, bromocriptine, placebo); df, degrees of freedom; Sens, ePAS sensitivity; time, pre- and post-stimulation (grand average over 30-minute post-stimulation); ηp^2^, partial eta-squared.

Bold *P* values indicate statistical significance.

### ePAS-Sensitivity-Dependent Effects of Applied Substances

**Figure 2.**
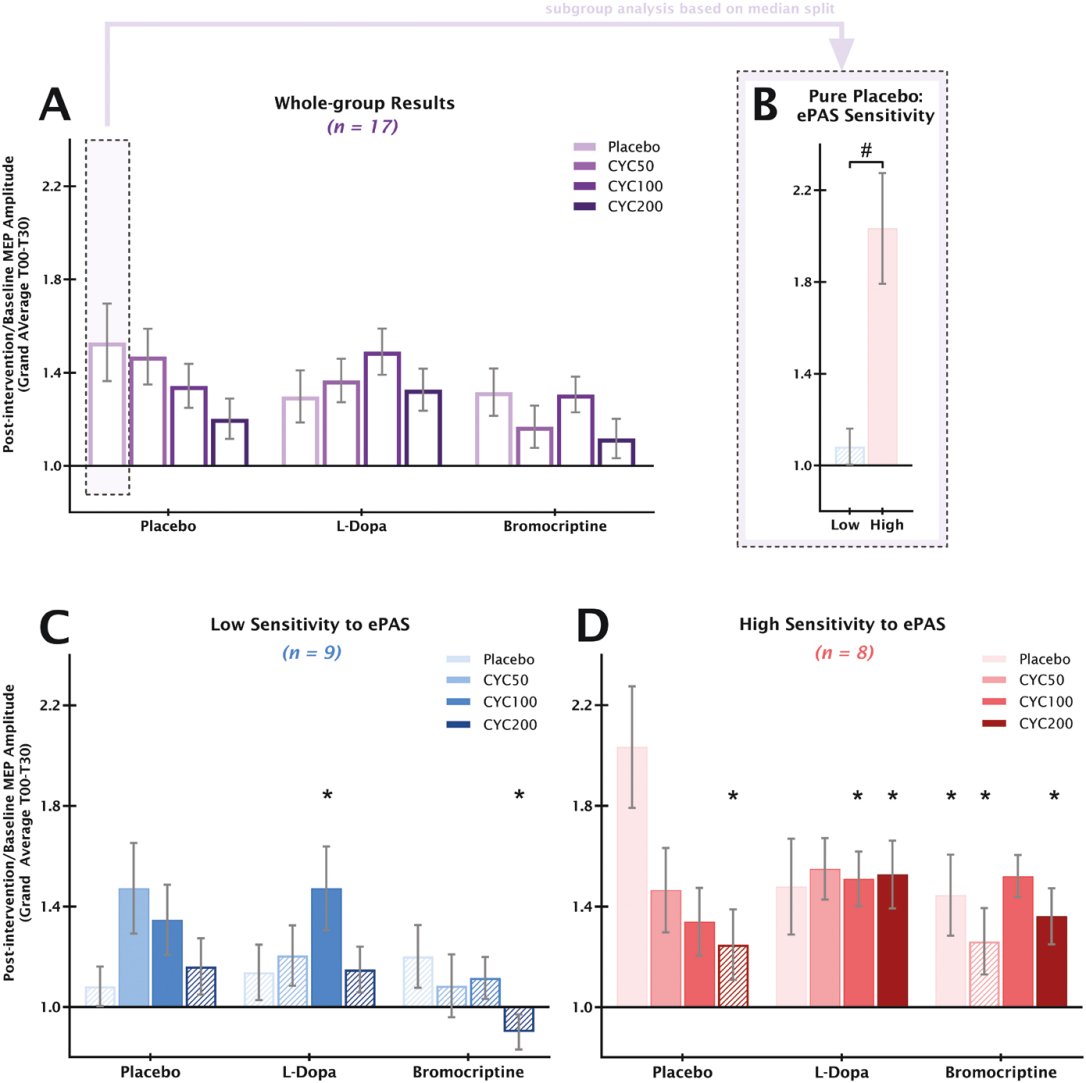
The baseline-standardized grand average (T00–T30 post-stimulation) of MEP amplitudes post-intervention across the whole sample and per sensitivity subgroup are shown. (A) Bars indicating baseline-standardized MEP amplitudes in each substance condition for all 17 participants grand-averaged over the after-measurements 0–30 minutes post-stimulation. (B) Based on high interindividual variability in response to ePAS observed in the pure placebo condition, the sample of 17 participants was median-split into low- and high-sensitivity subgroups. In the first 30 minutes after stimulation, ePAS induced LTP-like plasticity only in the high-sensitivity group. (C) In low-sensitivity participants, PAS combined with low and medium doses of CYC induced LTP-like plasticity, while under L-Dopa, only the medium CYC dose led to these effects. Regardless of CYC dose, ePAS failed to elicit facilitatory outcomes under the selective D2 receptor agonist bromocriptine. D: In high-sensitivity participants, both low and medium doses of CYC preserved LTP-like plasticity induced by PAS. Under DAergic agents, ePAS-induced facilitatory effects were largely preserved. Filled bars in B, C, and D indicate significant differences to the respective baseline of each intervention condition. In B, the hash symbol indicates a significant MEP difference between low- and high-sensitivity participants in the pure placebo condition (independent *t*-test, 2-tailed, #: *P <* .05). In C and D, asterisks indicate significant differences between the respective substance condition and the pure placebo substance condition of the respective sensitivity subgroup (paired *t*-tests, 2-tailed, *: *P <* .05). Error bars: SEM. CYC, D-Cycloserine; MEP, motor-evoked potential.

Under pure placebo, MEPs for low ePAS sensitivity did not differ from baseline and were significantly lower than for high ePAS sensitivity (*P* = .005, see Figure 2B and Table S12). MEPs in high-sensitivity participants were significantly increased compared to baseline (*P* = .004).

In low-sensitivity participants, low and medium doses of CYC significantly enhanced MEPs compared to baseline (low: *P* = .030; medium: *P* = .038; see Figure 2C), while high-dose CYC did not. With L-Dopa, ePAS had no effect on MEPs. Adding medium-dose CYC increased MEPs significantly compared to baseline (*P* = .022) and pure placebo (*P* = .023). This effect was absent for low- or high-dose CYC. Bromocriptine, alone or with any CYC dose, prevented ePAS-generated excitability enhancements in the low-sensitivity group. High-dose CYC reduced MEPs compared to pure placebo (*P* = .023) and to bromocriptine and placebo (*P* = .028), but these did not differ from baseline.

In high-sensitivity participants, low- and medium-dose CYC preserved MEP enhancement from ePAS (low-dose vs. baseline: *P* = .027; medium-dose vs. baseline: *P *= .040; see Figure 2D), while high-dose abolished it (vs. baseline: *P* = .120; vs. pure placebo: *P* = .030). Under L-Dopa, MEPs were significantly increased compared to baseline for L-Dopa alone and all CYC doses (alone: *P* = .040; low-dose: *P* = .003; medium-dose: *P* = .002; high-dose: *P* = .006). For medium- and high-dose CYC, MEPs were significantly smaller than for pure placebo (medium-dose: *P* = .034; high-dose: *P* = .026). Under selective D2 receptor enhancement, MEPs were enhanced for bromocriptine alone, and with medium- and high-dose CYC (alone: *P* = .028; medium-dose: *P* < .001; high-dose: *P* = .014). MEPs were significantly smaller than for pure placebo for bromocriptine alone (*P* = .041), for low-dose CYC (*P* = .030), and for high-dose CYC (*P* = .037).

## DISCUSSION

This study examined the contribution of D2 receptors to the focusing effect of DA on ePAS-induced LTP-like neuroplasticity, and its dependency on NMDA receptor activity. All participants tolerated ePAS well. Substances alone did not affect cortical excitability. Our main analysis across all participants did not show significant interaction effects between DAergic agents, CYC doses, and time. Splitting our sample into low- and high-sensitivity groups revealed that LTP-like effects were induced in high-sensitivity participants only. Adding small and medium doses of CYC induced excitatory plasticity in participants with low sensitivity and preserved effects for high sensitivity, while the high dose prevented LTP-like effects in both groups. DAergic modulation of neuroplasticity depended on ePAS sensitivity: In low-sensitivity participants, adding DAergic agents did not induce ePAS-generated plasticity, while in high-sensitivity participants, LTP-like effects were preserved for L-Dopa and diminished for bromocriptine. Medium-dose CYC induced ePAS-generated LTP-like plasticity under L-Dopa, while no CYC dose enhanced excitability under bromocriptine in low-sensitivity participants. In high-sensitivity participants, LTP-like plasticity was preserved for L-Dopa and all CYC doses, whereas under bromocriptine, only medium- and high-dose CYC preserved LTP-like effects.

### Sensitivity to ePAS

As reported in previous studies, participants showed high interindividual variability in response to ePAS in the present study.^10,25,26^ Strube et al.^10^ report that 47% of their participants showed post-stimulation MEPs exceeding 150% of baseline levels, comparable to 41% in our sample.

The reasons for this response variability might be manifold and are incompletely explored. Nitsche et al.^27^ report that PAS effects are sensitive to global neural activity in a brain circuitry. In line with a variable threshold for LTP-plasticity induction depending on postsynaptic activity levels,^28^ increased intracortical inhibition might impede plasticity induction by PAS.^29,30^ In accordance, the proportion of late I-waves elicited by TMS in PAS, which affects short-term intracortical inhibition, determines individual PAS response.^31^ Low- and high-sensitivity participants in this study might thus show different levels of intracortical inhibition, leading to divergent ePAS plasticity outcomes. PAS response also depends on genetics, as shown in a study on twins.^32^ One candidate genetic variation is the Val66Met polymorphism of the brain-derived neurotrophic factor, associated with reduced LTP-like induction by PAS.^33^ Moreover, variability of DAergic neurotransmission caused by genetics or other reasons might also play a role.^34–37^

### Dose-Dependent Effect of NMDA Receptor Activation on LTP-Like Plasticity

The effect of increased NMDA receptor activation on plasticity without additional DAergic receptor activation was nonlinear and dose-dependent in both ePAS sensitivity groups.

CYC affects neuroplasticity as a partial NMDA receptor agonist.^38^ Medium-dose CYC enhanced LTP-like neuroplasticity in visual and motor cortices and improved performance in incremental learning tasks.^39–41^ Under tDCS, medium-dose CYC prolonged LTP-like effects.^39^ The present study found discernible effects for low and high ePAS sensitivity: In the low-sensitivity group, LTP-like plasticity was only induced under medium and low doses of CYC, while ePAS-generated plasticity was largely preserved, but trendwise reduced, in the high-sensitivity group under identical dosages. These different result patterns in ePAS sensitivity groups might be caused by insufficient NMDA receptor activation in the low-sensitivity group, which is compensated for by CYC, while the same CYC dosages result in NMDA receptor over-activity in the high-sensitivity group, and thus might lead to counter-regulation and a gradual reduction of plasticity.^42^ In accordance with Ghanavati et al.,^13^ high-dose CYC abolished, or prevented LTP-like plasticity in the present study, potentially due to a shift from agonistic to antagonistic effects observed before for high-dose CYC.^43,44^

### DAergic Modulation Effects on ePAS-Induced LTP-Like Neuroplasticity

Both L-Dopa and bromocriptine alone preserved ePAS-induced LTP-like plasticity in the high-sensitivity group, although gradually reduced for selective D2 activation, which aligns with previous findings.^8,14^ ePAS in the low-sensitivity group did however not induce plasticity, and this was not altered by L-Dopa or bromocriptine. These PAS sensitivity-dependent effects suggest that interindividual heterogeneity in response to ePAS based on sample composition might account for partially heterogeneous results observed in previous studies involving L-Dopa.^4,8^

Adding NMDA receptor activation via CYC to DAergic agents led to ePAS sensitivity-dependent effects, while findings from both groups indicate a receptor subtype-dependency of DAergic effects on ePAS-generated plasticity. For the low-sensitivity group, plasticity was only induced under medium-dose CYC and L-Dopa, but not bromocriptine. An explanation for this result might be that L-Dopa does not only activate D2-like, but also D1-like receptor activity. The latter enhances NMDA receptor activity in plasticity induction scenarios.^45^ Moreover, increased D1 receptor activation via L-Dopa and D2 receptor block via sulpiride preserved ePAS-induced LTP-like plasticity in previous studies.^46,47^ Medium-dose CYC might have synergistic effects on this L-Dopa-driven NMDA receptor activity enhancement, while low- and high-dose CYC might not have this impact due to the reasons explained above. This D1 receptor-enhancing effect is absent for bromocriptine, resulting in an NMDA receptor activity-reducing effect solely driven by D2 receptors, which will abolish CYC effects. For the high ePAS-sensitivity group, we speculate that ePAS-generated NMDA receptor activity is strong enough to induce LTP-like plasticity, and to preserve it also under NMDA receptor activity reduction accomplished by L-Dopa, and bromocriptine. The gradual reduction of plasticity under both substances is likely caused by D2 receptor activation, which is supported by the trendwise larger plasticity reduction under the lowest CYC dose under bromocriptine. These mechanistic explanations are highly speculative at present and have to be substantiated in animal models and neuroimaging studies in humans.

### Functional Implications

One main finding of this study is increased knowledge about the mechanisms underlying the focusing effect of dopamine on LTP-like plasticity observed in previous studies, where it was demonstrated that DAergic agents abolish diffuse, but not focal LTP-like plasticity.^4^ Here it was shown that glutamatergic mechanisms are important for these effects, in line with similar results in a companion study, where DAergic effects on non-focal plasticity were explored.^13^ This further substantiates the crucial role of D2 receptors in the focusing effects of DA. Since neuroplasticity is the cellular basis of learning and memory formation, its DAergic modulation likely translates to cognitive performance (for a review, see Matzel and Sauce^48^). Papenberg et al.^37^ associated DA and D2 receptor availability with memory task performance, indicating that inhibitory D2 receptor-dependent mechanisms facilitate cognitive processing. The results of the present study do not propose a plasticity effect on cognitive performance per se, which is traditionally more associated with D1 receptors, but stresses the focusing effect of D2 receptors as a potential complementary candidate, which aligns with different task-dependent effects of DAergic modulation.^49-51^ Understanding DA’s role in modulating neuroplasticity and focusing cognitive processes is crucial for treating cognitive deficits in conditions with DAergic pathophysiology.^52-54^ In attention-deficit/hyperactivity disorder (ADHD), reduced D2 receptor availability is associated with motivation deficits and inattention,^55^ potentially in line with a weakened focusing effect due to DAergic imbalance. Meanwhile, D1 receptors contribute to memory formation and enhancement, and preservation of LTP-like plasticity. A selective D1 agonist has been shown to improve working memory performance in rats more effectively than traditional ADHD pharmacotherapy.^56^ Therefore, finding the optimal balance of D1, D2, and NMDA receptor activity through pharmacological augmentation could potentially restore relevant LTP-like effects from ePAS or tDCS, and improve cognitive symptoms, not only in ADHD, but also in other DA-associated conditions, such as schizophrenia. Moreover, we replicated the relatively large variability of ePAS-induced plasticity shown in previous studies, but also found that in the low-sensitivity subgroup, adding CYC in specific dosages enabled LTP-like plasticity in these participants. This finding might hold promise for future clinical application in stimulation-resistant patients and underlines the importance of individualizing treatment strategies when combining medication and brain stimulation.

### Limitations and Future Directions

Some limitations of this study should be considered. Blinding was not formally assessed; however, we are confident that neither participants nor the experimenter could identify individual substance conditions, as side effects were rare and occurred in a few subjects only. The post hoc median split into low- and high-sensitivity participants resulted in a small number of subjects per subgroup. Therefore, confirmatory studies with larger sample sizes are required. While our stimulation protocol started within the peak dose windows of the different substances derived from former studies, individual blood serum concentrations were not obtained and interindividual variability in pharmacokinetics cannot be ruled out. Our results were obtained from the left primary motor cortex serving as a model system, with MEPs as indirect output measures for cortical plasticity. Future studies should investigate whether these results can be replicated in other brain regions, or manifest in observable behavioral changes, considering that LTP, and LTP-like plasticity aligned with increased MEPs have been associated with improved motor learning and memory in previous studies.^57-59^ Finally, translating our results from healthy young participants to clinical populations with DA-associated disorders requires further investigation in the future.

## CONCLUSION

This study investigated NMDA-receptor-dependent effects of DAergic modulation on ePAS-induced neuroplasticity. We did not observe significant interactions between DAergic agents, CYC, and time across the entire sample. However, significant differences emerged based on sensitivity to ePAS, highlighting the need to further investigate how individual characteristics influence stimulation effects. In line with a focusing effect of DA, our findings suggest that optimal NMDA receptor activation is critical for ePAS-generated plasticity, with D2 receptor activity potentially reducing ePAS-induced LTP-like effects by diminishing NMDA receptor activity.

## Supplementary Material

pyaf038_suppl_Supplementary_Tables_S1-S12_Figure_S1

## Data Availability

The data underlying this article will be shared on reasonable request to the corresponding author.
