# Supplementary material for: NMDA receptor involvement in dopaminergic modulation of neuroplasticity induced by paired associative stimulation
Source: Int J Neuropsychopharmacol. 2025 May 30;28(6):pyaf038. doi: 10.1093/ijnp/pyaf038 (PMC12202998; doi:10.1093/ijnp/pyaf038)
Supplement: pyaf038_suppl_Supplementary_Tables_S1-S12_Figure_S1 [file pyaf038_suppl_supplementary_tables_s1-s12_figure_s1.docx]

# Supplement

**Table S1**

*Grand average per participant for MEPs obtained within the time windows from 0-30, and 0-120 min after stimulation in the pure placebo condition*

| **Low sensitivity** | |  | **High sensitivity** | |  |
| --- | --- | --- | --- | --- | --- |
| Participant | T00-T30 | T00-T120 | Participant | T00-T30 | T00-T120 |
| 1 | 0.520 | 0.615 | 10 | 1.297 | 1.378 |
| 2 | 0.929 | 1.013 | 11 | 1.603 | 1.529 |
| 3 | 1.045 | 1.018 | 12 | 1.614 | 1.548 |
| 4 | 1.177 | 1.146 | 13 | 1.772 | 1.654 |
| 5 | 1.179 | 1.228 | 14 | 1.892 | 1.754 |
| 6 | 1.180 | 1.194 | 15 | 1.902 | 1.640 |
| 7 | 1.184 | 1.231 | 16 | 3.095 | 2.998 |
| 8 | 1.245 | 1.381 | 17 | 3.097 | 3.141 |
| 9 | 1.278 | 1.250 |  |  |  |
| Mean (SD) | 1.082  (0.236) | 1.120  (0.221) |  | 3.097  (0.683) | 1.955  (0.697) |

**Table S2**

*Intensity ratings of medication side effects per experimental condition*

|  | **Sleepiness** | **Dizziness** | **Headache** | **Nausea** | **Heart palp.** |
| --- | --- | --- | --- | --- | --- |
| PLC + PLC | 0 (0) | 0 (0) | 0 (0) | 0 (0) | 0 (0) |
| PLC + 50 mg CYC | 0 (0) | 0 (0) | 0 (0) | 0 (0) | 0 (0) |
| PLC + 100 mg CYC | 0 (0) | 0 (0) | 0.06 (0.24) | 0 (0) | 0 (0) |
| PLC + 200 mg CYC | 0.18 (0.73) | 0 (0) | 0.06 (0.24) | 0 (0) | 0 (0) |
| L-Dopa + PLC | 0.12 (0.49) | 0.06 (0.24) | 0.06 (0.24) | 0.24 (0.97) | 0.06 (0.24) |
| L-Dopa + 50 mg CYC | 0 (0) | 0 (0) | 0 (0) | 0 (0) | 0 (0) |
| L-Dopa + 100 mg CYC | 0.35 (1.00) | 0 (0) | 0 (0) | 0 (0) | 0 (0) |
| L-Dopa + 200 mg CYC | 0 (0) | 0 (0) | 0 (0) | 0 (0) | 0 (0) |
| Bromo + PLC | 0.35 (1.06) | 0.12 (0.49) | 0.06 (0.24) | 0.12 (0.33) | 0.18 (0.73) |
| Bromo + 50 mg CYC | 0.29 (0.85) | 0.24 (0.97) | 0.12 (0.33) | 0.06 (0.24) | 0.18 (0.53) |
| Bromo + 100 mg CYC | 0.41 (1.18) | 0.18 (0.73) | 0 (0) | 0.24 (0.75) | 0 (0) |
| Bromo + 200 mg CYC | 0.24 (0.75) | 0.41 (1.18) | 0.18 (0.53) | 0.59 (1.23) | 0.06 (0.24) |

*Note.* Ratings were given on a scale from 0 (‘absent’) to 4 (‘very strong’). Data are presented as mean (SD). Palp.: palpitations, PLC: placebo, Bromo: bromocriptine, CYC: D-cycloserine.

**Table S3**

*Intensity ratings of stimulation side effects per experimental condition*

|  | **Headache** | **Neck Pain** | **Concen-tration Diff.** | **Skin Irritations** | **Sleepiness** |
| --- | --- | --- | --- | --- | --- |
| PLC + PLC | 0.18 (0.24) | 0 (0) | 0 (0) | 0 (0) | 0.18 (0.53) |
| PLC + 50 mg CYC | 0 (0) | 0 (0) | 0 (0) | 0 (0) | 0.06 (0.24) |
| PLC + 100 mg CYC | 0.18 (0.39) | 0.06 (0.24) | 0 (0) | 0 (0) | 0.06 (0.24) |
| PLC + 200 mg CYC | 0.06 (0.24) | 0 (0) | 0 (0) | 0 (0) | 0 (0) |
| L-Dopa + PLC | 0.06 (0.24) | 0 (0) | 0 (0) | 0 (0) | 0 (0) |
| L-Dopa + 50 mg CYC | 0.06 (0.24) | 0 (0) | 0 (0) | 0 (0) | 0.06 (0.24) |
| L-Dopa + 100 mg CYC | 0.06 (0.24) | 0 (0) | 0 (0) | 0 (0) | 0.06 (0.24) |
| L-Dopa + 200 mg CYC | 0.12 (0.33) | 0 (0) | 0 (0) | 0 (0) | 0 (0) |
| Bromo + PLC | 0.18 (0.53) | 0 (0) | 0 (0) | 0.06 (0.24) | 0.18 (0.53) |
| Bromo + 50 mg CYC | 0.06 (0.24) | 0 (0) | 0.06 (0.24) | 0 (0) | 0.12 (0.33) |
| Bromo + 100 mg CYC | 0 (0) | 0 (0) | 0 (0) | 0 (0) | 0.12 (0.33) |
| Bromo + 200 mg CYC | 0.18 (0.53) | 0 (0) | 0 (0) | 0 (0) | 0.18 (0.53) |

*Note.* Ratings were given on a scale from 0 (‘absent’) to 4 (‘very strong’). Data are presented as mean (SD). Diff: Difficulties, PLC: placebo, CYC: D-cycloserine, Bromo: bromocriptine.

**Table S4**

*Results of the one-way repeated-measures ANOVAs for adverse effects from substances between conditions*

|  | ***df*** | ***F*** | ***p*** | ***η^2^_p_*** |
| --- | --- | --- | --- | --- |
| Sleepiness | 11, 192 | 0.975 | .470 | .053 |
| Dizziness | 11, 192 | 1.120 | .348 | .060 |
| Headache | 11, 192 | 1.052 | .402 | .057 |
| Nausea | 11, 192 | 2.021 | **.028** | .104 |
| Heart palpitations | 11, 192 | 1.060 | .434 | .055 |

*Note*. df: degrees of freedom, η_p_ ^2^: partial eta squared. Significances are marked in bold.

**Table S5**

*Results of the one-way repeated-measures ANOVA for adverse effects from stimulation between conditions*

|  | ***df*** | ***F*** | ***p*** | ***η^2^_p_*** |
| --- | --- | --- | --- | --- |
| Headache | 11, 192 | 0.685 | .752 | 0.038 |
| Neck pain | 11, 192 | 1.000 | .448 | 0.054 |
| Concentration difficulties | 11, 192 | 0.909 | .533 | 0.050 |
| Skin irritations | 11, 192 | 1.000 | .448 | 0.054 |
| Sleepiness | 11, 192 | 0.740 | .699 | 0.041 |

*Note*. df: degrees of freedom, η_p_ ^2^: partial eta squared.

**Table S6**

*Results of the two-way repeated-measures ANOVA with the within-subject factors condition (all experimental conditions) and time (baseline 1 and baseline 2/3) on MEP amplitudes and stimulation intensities*

|  | ***df*** | ***F*** | ***p*** | ***η_p_^2^*** |
| --- | --- | --- | --- | --- |
| **MEP amplitudes (BL1 and BL2)** |  |  |  |  |
| Condition | 11, 176 | 0.907 | .535 | .054 |
| Time | 1, 16 | 0.433 | .520 | .026 |
| Condition x Time | 11, 176 | 0.925 | .518 | .055 |
|  |  |  |  |  |
| **Stimulation intensities (BL1 and BL3)** |  |  |  |  |
| Condition | 4.7, 74.4 | 0.517 | .750 | .031 |
| Time | 1, 16 | 3.071 | .056 | .210 |
| Condition x Time | 11, 176 | 1.176 | .307 | .068 |

*Note.* BL: baseline, df: degrees of freedom, ηp^2^: partial eta squared.


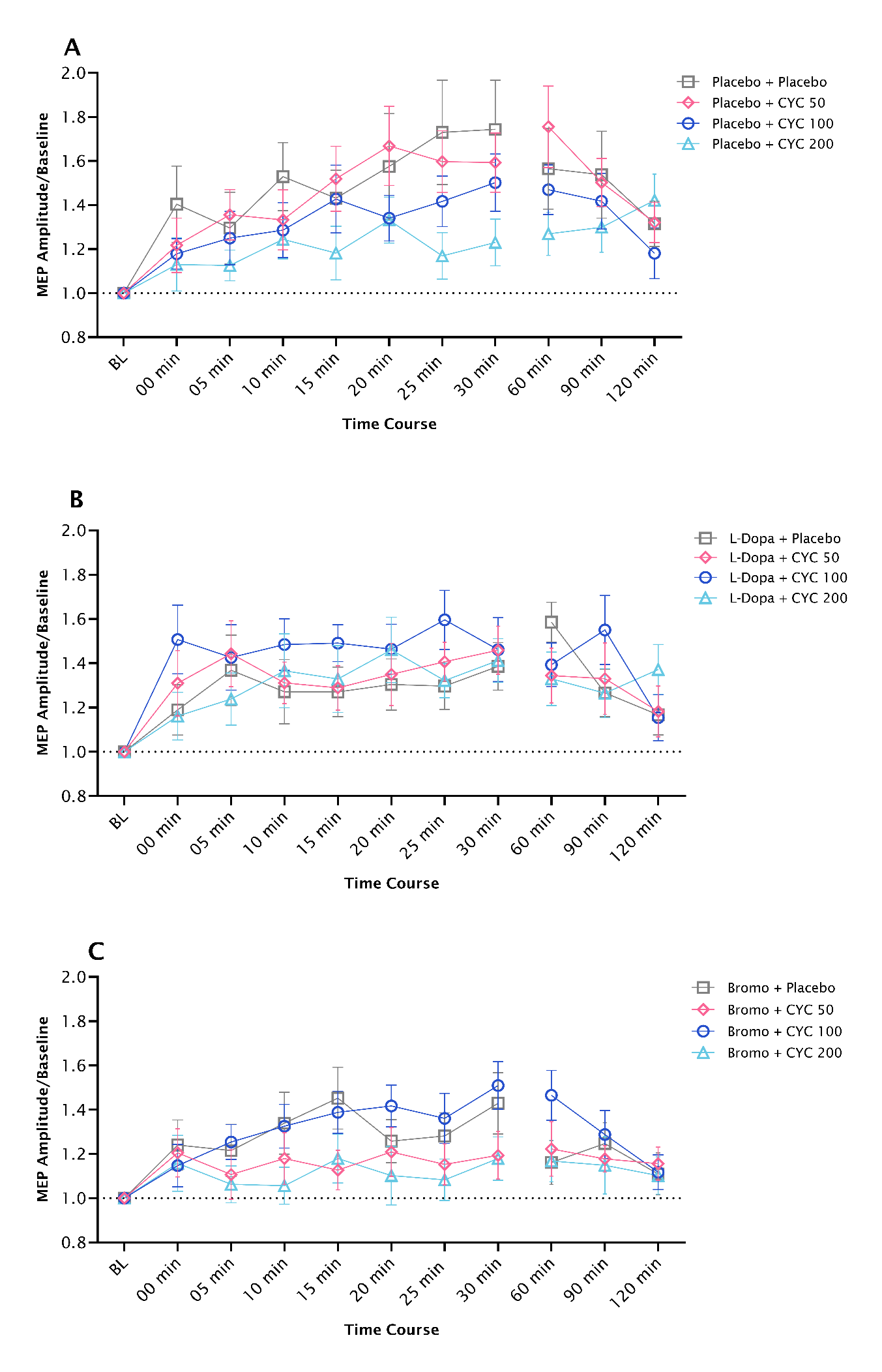


**Figure S1.** Whole-group MEP responses over time per experimental conditions. No significant interactions for DAergic agents, CYC doses and time on the whole-group level were revealed. A) Placebo and CYC doses, B) L-Dopa and CYC doses, C) Bromocriptine and CYC doses. Error bars: SEM. Baseline (BL) and time points of measurements after plasticity induction are displayed on the x-axis, mean MEP amplitudes post-intervention normalized to baseline on the y-axis. Bromo: bromocriptine, CYC: D-cycloserine.

**Table S7**

*Results of the four-way mixed-design ANOVA (DA x CYC x time x gender)*

|  | ***df*** | ***F*** | ***p*** | ***η_p_^2^*** |
| --- | --- | --- | --- | --- |
| Gender | 1, 12 | 0.459 | .511 | .037 |
| DA | 2, 24 | 0.118 | .889 | .010 |
| CYC | 3, 36 | 0.716 | .549 | .056 |
| Time | 2.44, 29.33 | 0.779 | .492 | .061 |
| DA x gender | 2, 24 | 1.259 | .302 | .095 |
| CYC x gender | 3, 36 | 0.559 | .646 | .044 |
| Time x gender | 2.44, 29.33 | 1.869 | .165 | .135 |
| DA x CYC | 3.29, 39.45 | 1.315 | .283 | .099 |
| DA x time | 6.51, 78.09 | 0.404 | .887 | .033 |
| CYC x time | 7.58. 90.90 | 1.781 | .095 | .129 |
| DA x CYC x gender | 3.29, 39.45 | 1.322 | .280 | .099 |
| DA x time x gender | 6.51, 78.09 | 0.638 | .712 | .051 |
| CYC x time x gender | 7.58. 90.90 | 0.570 | .791 | .045 |
| DA x CYC x time | 8.08, 96.91 | 1.581 | .140 | .116 |
| DA x CYC x time x gender | 8.08, 96.91 | 1.028 | .421 | .079 |

*Note.* DA: dopaminergic agents (L-Dopa, bromocriptine, placebo), CYC: D-cycloserine (50, 100, and 200 mg, placebo), time: all 11 after-measurements post-stimulation. df: degrees of freedom, ηp^2^: partial eta squared.

**Table S8**

*Results of the one-way repeated-measures ANOVA for stimulation intensities and baseline MEPs*

|  | ***df*** | ***F*** | ***p*** | ***η_p_^2^*** |
| --- | --- | --- | --- | --- |
| **Low sensitivity** |  |  |  |  |
| TMS intensity BL1 | 11, 96 | 0.015 | 1.000 | .002 |
| TMS intensity BL3 | 11, 96 | 0.018 | 1.000 | .002 |
| Peripheral stimulation intensity | 11, 96 | 0.088 | 1.000 | .010 |
| MEP BL1 | 11, 96 | 0.688 | .747 | .073 |
| MEP Bl 2 | 11, 96 | 0.480 | .911 | .052 |
|  |  |  |  |  |
| **High sensitivity** |  |  |  |  |
| TMS intensity BL1 | 11, 84 | 0.025 | 1.000 | .003 |
| TMS intensity BL3 | 11, 84 | 0.045 | 1.000 | .006 |
| Peripheral stimulation intensity | 11, 84 | 0.329 | .977 | .041 |
| MEP BL1 | 11, 84 | 0.414 | .946 | .051 |
| MEP BL2 | 11, 84 | 0.750 | .688 | .089 |

*Note.* df: degrees of freedom, ηp^2^: partial eta squared, BL: baseline.

**Table S9**

*Results of the two-way repeated-measures ANOVA with the within-subject factors condition (all experimental conditions) and time (baseline 1 and baseline 3) on stimulation intensities*

|  | ***df*** | ***F*** | ***p*** | ***η_p_^2^*** |
| --- | --- | --- | --- | --- |
| **Low sensitivity** |  |  |  |  |
| Condition | 3.6, 28.9 | 0.709 | .579 | .081 |
| Time | 1, 8 | 0.837 | .387 | .095 |
| Condition x Time | 4.6, 36.6 | 1.242 | .310 | .134 |
|  |  |  |  |  |
| **High sensitivity** |  |  |  |  |
| Condition | 4.2, 29.1 | 1.624 | .191 | .189 |
| Time | 1, 7 | 3.596 | .100 | .339 |
| Condition x Time | 3.0, 20.7 | 1.165 | .347 | .143 |

*Note.* df: degrees of freedom, ηp^2^: partial eta squared.

**Table S10**

*Results of the two-way repeated-measures ANOVA with the within-subject factors condition (all experimental conditions) and time (baseline 1 and baseline 2) on MEP amplitudes*

|  | ***df*** | ***F*** | ***p*** | ***η_p_^2^*** |
| --- | --- | --- | --- | --- |
| **Low sensitivity** |  |  |  |  |
| Condition | 3.8, 30.6 | 1.434 | .248 | .152 |
| Time | 1, 8 | 0.142 | .716 | .017 |
| Condition x Time | 4.2, 33.5 | 0.725 | .587 | .083 |
|  |  |  |  |  |
| **High sensitivity** |  |  |  |  |
| Condition | 3.4, 23.5 | 0.834 | .500 | .106 |
| Time | 1, 7 | 1.132 | .323 | .139 |
| Condition x Time | 4.1, 28.6 | 0.877 | .492 | .111 |

*Note.* df: degrees of freedom, ηp^2^: partial eta squared.

**Table S11**

*Results of the independent t-tests conducted on averaged baseline parameters between low- and high-sensitivity individuals*

|  | **Mean**  **low sens.** | **Mean**  **high sens.** | ***df*** | ***t*** | ***p*** | ***Cohen’s d*** |
| --- | --- | --- | --- | --- | --- | --- |
| TMS intensity BL1 | 53.361 | 57.229 | 15 | -0.703 | .493 | 0.356 |
| TMS intensity BL3 | 53.440 | 57.509 | 15 | -0.733 | .475 | -0.342 |
| Peri. stim. intensity | 66.972 | 62.521 | 15 | 0.732 | .475 | -0.356 |
| MEP BL1 | 1.004 | 1.004 | 15 | -0.003 | .998 | -0.002 |
| MEP BL2 | 1.010 | 0.972 | 15 | 0.967 | .349 | 0.470 |

*Note.* sens: sensitivity, df: degrees of freedom, BL: baseline, peri stim: peripheral stimulation.

**Table S12**

*Results of the paired t-tests conducted on grand-averaged data (T00-T30) for low- and high-sensitivity participant groups: Differences between experimental conditions and baseline, experimental conditions and pure placebo, and DAergic placebo conditions and CYC doses*

| **Comparison** | **Mean 1** | **Mean 2** | ***df*** | ***t*** | ***p*** | **Cohen’s *d*** |
| --- | --- | --- | --- | --- | --- | --- |
| **Low sensitivity** |  |  |  |  |  |  |
| Baseline vs. PLCPLC | 1 | 1.082 | 8 | 1.043 | .328 | 0.348 |
| Baseline vs. PLCCYC50 | 1 | 1.473 | 8 | 2.624 | **.030** | 0.875 |
| Baseline vs. PLCCYC100 | 1 | 1.347 | 8 | 2.477 | **.038** | 0.826 |
| Baseline vs. PLCCYC200 | 1 | 1.161 | 8 | 1.435 | .189 | 0.478 |
| Baseline vs. LDPLC | 1 | 1.138 | 8 | 1.249 | .247 | 0.416 |
| Baseline vs. LDCYC50 | 1 | 1.205 | 8 | 1.705 | **.022** | 0.568 |
| Baseline vs. LDCYC100 | 1 | 1.472 | 8 | 2.836 | .141 | 0.945 |
| Baseline vs. LDCYC200 | 1 | 1.149 | 8 | 1.634 | .247 | 0.545 |
| Baseline vs. BRPLC | 1 | 1.201 | 8 | 1.612 | .146 | 0.537 |
| Baseline vs. BRCYC50 | 1 | 1.084 | 8 | 0.673 | .520 | 0.224 |
| Baseline vs. BRCYC100 | 1 | 1.116 | 8 | 1.387 | .203 | 0.462 |
| Baseline vs. BRCYC200 | 1 | 0.900 | 8 | -1.425 | .192 | -0.475 |
| PLCPLC vs. PLCCYC50 | 1.082 | 1.473 | 8 | -1.895 | .095 | -0.632 |
| PLCPLC vs. PLCCYC100 | 1.082 | 1.347 | 8 | -1.834 | .104 | -0.611 |
| PLCPLC vs. PLCCYC200 | 1.082 | 1.161 | 8 | -0.801 | .446 | -0.267 |
| PLCPLC vs. LDPLC | 1.082 | 1.138 | 8 | -0.595 | .569 | -0.198 |
| PLCPLC vs. LDCYC50 | 1.082 | 1.205 | 8 | -1.079 | .312 | -0.360 |
| PLCPLC vs. LDCYC100 | 1.082 | 1.472 | 8 | -2.819 | **.023** | -0.940 |
| PLCPLC vs. LDCYC200 | 1.082 | 1.149 | 8 | -1.635 | .141 | -0.545 |
| PLCPLC vs. BRPLC | 1.082 | 1.201 | 8 | -0.963 | .364 | -0.321 |
| PLCPLC vs. BRCYC50 | 1.082 | 1.084 | 8 | -0.015 | .989 | -0.005 |
| PLCPLC vs. BRCYC100 | 1.082 | 1.116 | 8 | -0.422 | .684 | -0.141 |
| PLCPLC vs. BRCYC200 | 1.082 | 0.900 | 8 | 2.796 | **.023** | 0.932 |
| LDPLC vs. LDCYC50 | 1.138 | 1.205 | 8 | -0.481 | .643 | -0.160 |
| LDPLC vs. LDCYC100 | 1.138 | 1.472 | 8 | -2.249 | .055 | -0.750 |
| LDPLC vs. LDCYC200 | 1.138 | 1.149 | 8 | -1.54 | .882 | -0.051 |
| BRPLC vs. BRCYC50 | 1.201 | 1.084 | 8 | 0.899 | .395 | 0.300 |
| BRPLC vs. BRCYC100 | 1.201 | 1.116 | 8 | 0.599 | .566 | 0.200 |
| BRPLC vs. BRCYC200 | 1.201 | 0.900 | 8 | 2.674 | **.028** | 0.891 |
| **High sensitivity** |  |  |  |  |  |  |
| PLCPLC: low vs. high sens | 1.082 | 2.034 | 8.476 | -3.747 | **.005** | -1.914 |
| Baseline vs. PLCPLC | 1 | 2.034 | 7 | 4.280 | **.004** | 1.513 |
| Baseline vs. PLCCYC50 | 1 | 1.465 | 7 | 2.777 | **.027** | 0.982 |
| Baseline vs. PLCCYC100 | 1 | 1.339 | 7 | 2.519 | **.040** | 0.891 |
| Baseline vs. PLCCYC200 | 1 | 1.248 | 7 | 1.771 | .120 | 0.626 |
| Baseline vs. LDPLC | 1 | 1.479 | 7 | 2.518 | **.040** | 0.890 |
| Baseline vs. LDCYC50 | 1 | 1.549 | 7 | 4.502 | **.003** | 1.592 |
| Baseline vs. LDCYC100 | 1 | 1.510 | 7 | 4.681 | **.002** | 1.655 |
| Baseline vs. LDCYC200 | 1 | 1.527 | 7 | 3.903 | **.006** | 1.380 |
| Baseline vs. BRPLC | 1 | 1.446 | 7 | 2.765 | **.028** | 0.978 |
| Baseline vs. BRCYC50 | 1 | 1.262 | 7 | 1.979 | .088 | 0.700 |
| Baseline vs. BRCYC100 | 1 | 1.521 | 7 | 6.255 | **< .001** | 2.212 |
| Baseline vs. BRCYC200 | 1 | 1.361 | 7 | 3.247 | **.014** | 1.148 |
| PLCPLC vs. PLCCYC50 | 2.034 | 1.465 | 7 | 1.939 | .094 | 0.686 |
| PLCPLC vs. PLCCYC100 | 2.034 | 1.339 | 7 | 2.207 | .063 | 0.780 |
| PLCPLC vs. PLCCYC200 | 2.034 | 1.248 | 7 | 2.705 | **.030** | 0.956 |
| PLCPLC vs. LDPLC | 2.034 | 1.479 | 7 | 1.928 | .095 | 0.682 |
| PLCPLC vs. LDCYC50 | 2.034 | 1.549 | 7 | 1.549 | .165 | 0.548 |
| PLCPLC vs. LDCYC100 | 2.034 | 1.510 | 7 | 2.629 | **.034** | 0.930 |
| PLCPLC vs. LDCYC200 | 2.034 | 1.527 | 7 | 2.825 | **.026** | 0.999 |
| PLCPLC vs. BRPLC | 2.034 | 1.446 | 7 | 2.501 | **.041** | 0.884 |
| PLCPLC vs. BRCYC50 | 2.034 | 1.262 | 7 | 2.722 | **.030** | 0.963 |
| PLCPLC vs. BRCYC100 | 2.034 | 1.521 | 7 | 1.715 | .130 | 0.606 |
| PLCPLC vs. BRCYC200 | 2.034 | 1.361 | 7 | 2.576 | **.037** | 0.911 |
| LDPLC vs. LDCYC50 | 1.479 | 1.549 | 7 | -0.274 | .792 | -0.097 |
| LDPLC vs. LDCYC100 | 1.479 | 1.510 | 7 | -0.133 | .898 | -0.047 |
| LDPLC vs. LDCYC200 | 1.479 | 1.527 | 7 | -0.330 | .751 | -0.117 |
| BRPLC vs. BRCYC50 | 1.446 | 1.262 | 7 | 1.598 | .154 | 0.565 |
| BRPLC vs. BRCYC100 | 1.446 | 1.521 | 7 | -0.366 | .725 | -0.129 |
| BRPLC vs. BRCYC200 | 1.446 | 1.361 | 7 | 0.515 | .622 | 0.182 |

*Note.* PLC: placebo, LD: L-Dopa, BR: bromocriptine, CYC: D-cycloserine, *df:* degrees of freedom, sens: sensitivity. Significances are marked in bold.
